# Enantiomeric Separation and Determination of the Enantiomeric Impurity of Armodafinil by Capillary Electrophoresis with Sulfobutyl Ether-β-cyclodextrin as Chiral Selector

**DOI:** 10.3390/molecules17010303

**Published:** 2011-12-30

**Authors:** Wei Wang, Suyun Xiang, Xiaojuan Zhou, Yibing Ji, Bingren Xiang

**Affiliations:** 1 Department of Analytical Chemistry, China Pharmaceutical University, Nanjing 211198, China; 2 College of Life Science, Nanjing Normal University, Nanjing 210046, China; 3 Key Laboratory of Drug Quality Control and Pharmacovigilance (Ministry of Education), China Pharmaceutical University, Nanjing 210009, China; 4 Center for Instrument Analysis, China Pharmaceutical University, Nanjing, 210009, China

**Keywords:** capillary electrophoresis, armodafinil, enantiomeric impurity, sulfobutyl ether-β-cyclodextrin

## Abstract

A selective capillary electrophoresis method using sulfobutyl ether-β-cyclodextrin as a chiral selector was developed and validated for the determination of the enantiomeric impurity of (R)-modafinil, *i.e.*, armodafinil. Several parameters were optimized for a satisfactory enantioresolution, including the type and concentration of chiral selector and organic modifier, pH of background electrolyte (BGE), capillary temperature. The finally adopted condition was: 20 mmol/L phosphate buffer at pH 7.5, containing 20 mmol/L sulfobutyl ether-β-cyclodextrin and 20% methanol, at temperature of 25 °C. A good resolution of 3.3 for the two enantiomers of modafinil was achieved by applying the optimal conditions. The limit of detection (LOD) and limit of quantification (LOQ) of (S)-modafinil were 1.25 μg/mL and 2.50 μg/mL, respectively. The established method was also proven to display good selectivity, repeatability, linearity and accuracy. Finally, the method was used to investigate the enantiomeric purity of armodafinil in bulk samples.

## 1. Introduction

**Figure 1:**
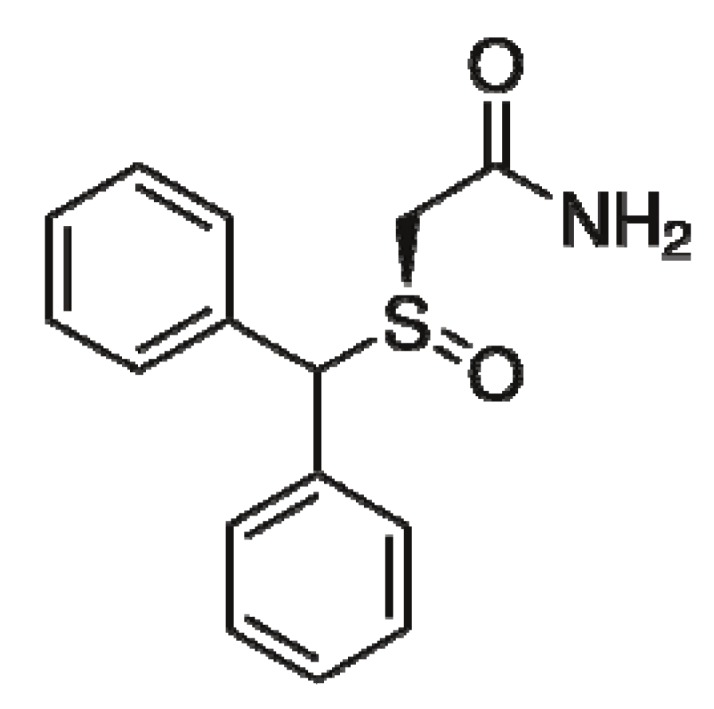
Chemical structure of armodafinil.

Modafinil is an analeptic drug for the treatment of excessive sleepiness caused by narcolepsy, shift work sleep disorder and obstructive sleep apnea [1,2,3]. Compared with other stimulants, it has a low potential risk of dependence and tolerance, and few serious side effects as well [4,5]. Modafinil is a chiral compound due to the presence of asymmetric sulfoxide function, but it is administrated as a racemic mixture of its (*R*)- and (*S*)-enantiomers. Both enantiomers are pharmacologically active; however, pharmacological studies showed that the two enantiomers differ in their pharmacological properties. The (*R*)-enantiomer, also known as armodafinil [(−)-2-[(*R*)-(diphenylmethyl)-sulfinyl]acetamide, Figure 1], is reported to has a longer half-life than the (*S*)-enantiomer and the proportion of circulating (*R*)-modafinil has been found to be as much as 3-fold that of circulating (*S*)-modafinil [6,7,8]. Due the significant pharmacokinetic difference between the two enantiomers, armodafinil was produced by the pharmaceutical company Cephalon Inc. and approved by the FDA in 2007 for the treatment of narcolepsy and shift work sleep disorder, and as an adjunctive treatment for obstructive sleep apnea [9,10]. Furthermore, it has undergone clinical trials evaluating it as a treatment for serious medical conditions such as bipolar depression and fatigue in conditions such as Parkinson's disease and cancer [11]. According to the ICH guidance entitled “*Specifications: Test procedures and acceptance criteria for new drug substances and new drug products: Chemical substances*” [12], for chiral drug substances which are developed as a single enantiomer, control of the other enantiomer should be considered in the same manner as for other impurities. Therefore, it is important to establish an enantiomeric separation and determination method for the control of enantiomeric impurity of armodafinil. 

Several studies have been reported for the enantioseparation and quantitative analysis of modafinil in biological matrix or formulation [13,14,15,16,17]. However in most of these reported methods, the enantioresolution of modafinil was achieved by using expensive chiral columns. To the best of our knowledge, there is no reported work concerning the quality control of the (*S*)-enantiomer impurity of armodafinil.

In recent years, capillary electrophoresis (CE) has been widely used for enantiomer separation [18,19,20,21] and enantiomeric impurity determination [22,23,24,25,26] thanks to its high efficiency, rapid analysis and low cost of operation. Enantioresolution for CE is usually achieved by adding chiral selectors to the buffer. Versatile native and modified cyclodextrins (CDs) are one of the most commonly used chiral selectors for the inclusion of solute hydrophobic portions by the cavity of CDs and the weak interactions of enantiomers with CDs (such as hydrophobic interactions, hydrogen bonding and van der Waals force, *etc.*) leading to differences in stability constants of temporary diastereomeric complexes. Besides, the differences in hydrodynamic resistance arising from the differences in shape, size or hydrodynamic radii, *etc.*, will result in distinction of complexes mobility. Thus, the differences in stability constants or mobility of complexes in CE will allow the chiral discrimination [27,28,29,30]. Consequently this work was devoted to establishing a CE method for the enantiomeric separation and determination of the enantiomeric impurity of armodafinil with CDs as chiral selector.

## 2. Results and Discussion

### 2.1. Optimization of Enantiomeric Separation

Several native and derivatized CDs, namely β-CD, γ-CD, hydroxypropyl-β-CD (HP-β-CD), methyl-β-CD (M-β-CD), hydroxypropyl-γ-CD (HP-γ-CD) and sulfobutyl ether-β-CD (SBE_7_-β-CD) were investigated at a concentration of 20 mmol/L. However, only SBE_7_-β-CD was able to separate the modafinil enantiomers as revealed by initial experiments. The reason maybe that modafinil enantiomers are neutral compounds (pKa 19.25) which could hardly be separated by CE with neutral selectors, but SBE_7_-β-CD is easily charged and the charged enantiomer-selector complexes will not co-migrate with the electroosmotic flow (EOF). Thus, the differences in equilibrium constants or mobility of complexes will be helpful to the enantioseparation of enantiomers. Therefore, SBE_7_-β-CD was used as chiral selector for further studies and a more exhaustive study was performed on the influence of several parameters on the enantioresolution of the modafinil enantiomers. 

#### 2.1.1. Effects of Organic Modifier

**Figure 2:**
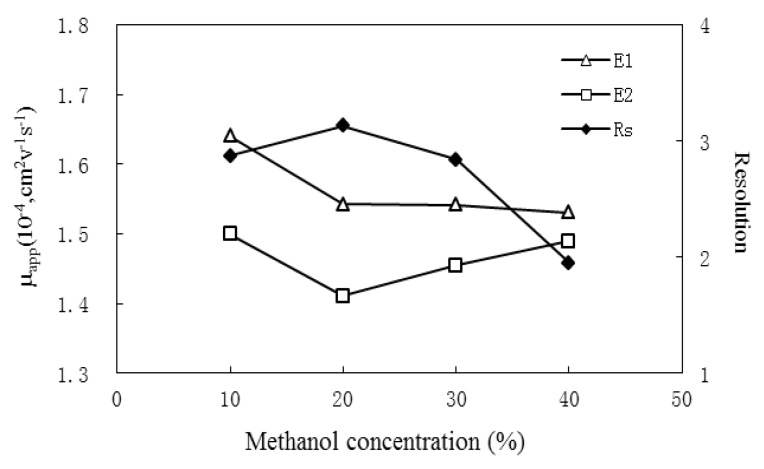
The effect of methanol concentration on resolution. Separation condition: 20 mmol/L SBE_7_-β-CD in 20 mmol/L phosphate buffer (pH 7.0); temperature, 25 °C; applied voltage, 20 kV; detection wavelength, 225 nm; injection, 50 mbar for 5 s; capillary, 50 cm × 50 μm i.d. with effective length of 41.5 cm.

The organic modifier is one of the factors often investigated in CE analysis, especially for medium or small polar compounds. In addition to improving the solubility of analyte, organic modifiers can alter the viscosity, ionic strength and the hydrophobicity of the background electrolyte (BGE), and accordingly, affect the interaction between enantiomers and chiral selector. The effects of modifier type and concentration on enantioresolution were studied. Three organic additives methanol, acetonitrile and isopropanol were each added to the BGE to find the optimal one. The comparative results indicated that methanol is the most effective one considering the peak shapes and the resolution of enantiomers. Subsequently, the effects of methanol concentration on resolution were studied by varying the percentage from 10% to 40% (v/v). The result (Figure 2) indicates that an optimal resolution was obtained when 20% methanol added to the BGE. This reason might be that the improvement of BGE viscosity along with the increase of methanol concentration results in a longer migration time and longer interaction time of SBE_7_-β-CD with the enantiomers, and thus, the resolution was observed to get better when methanol ascended from 10% to 20%. However, higher concentrations of methanol (20–40%) might be unfavorable for the inclusion of CDs and analytes, and accordingly, lead to poorer resolution.

#### 2.1.2. Effects of pH

The pH of buffer plays an important role in the enantiomeric separation process. In this study, the influence of pH was investigated with the tested pH values controlled at 6.0, 6.5, 7.0, 7.5 and 8.0. The results lead to the conclusion that the apparent electrophoretic mobility (*µ_app_*) increases from pH 6.0 to pH 6.5 for the EOF generally increases with the pH. However, when the pH increases from 7.0 to 8.0, the chiral selector, SBE_7_-β-CD, is negatively charged and migrates to the anode relatively, accordingly decelerates the selector-analyte complex, that is, the *µ_app_* of the analyte decreases.

**Figure 3:**
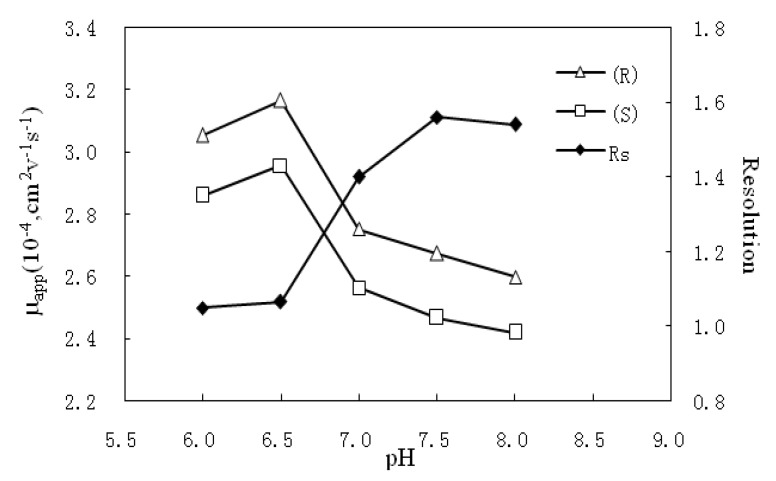
The effect of pH on resolution. Separation condition: 5 mmol/L SBE_7_-β-CD in 20 mmol/L phosphate buffer containing 20% methanol; the other conditions are same as in Figure 2.

Besides, the buffer pH will affect the inclusion constant and the interaction time of the chiral selector and analyte. The pH effect on resolution is a comprehensive result of these aspects. The result shown in Figure 3 reveals that the resolution tended to increase with the buffer pH varying from 6.0 to 7.5, and then became relatively flat above pH 7.5. An optimal enantioresolution of 1.56 was obtained at a pH at 7.5.

#### 2.1.3. Effects of SBE_7_-β-CD Concentration

**Figure 4:**
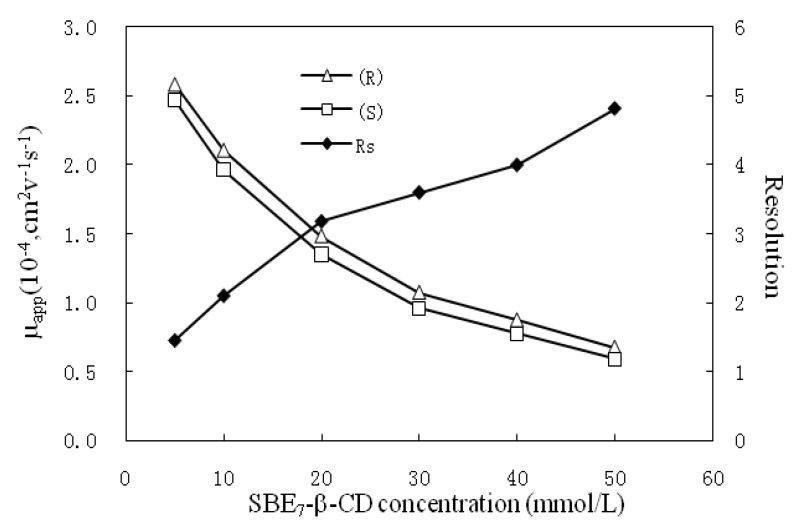
The effect of SBE_7_-β-CD concentration on resolution. Separation condition: 20 mmol/L phosphate buffer at pH 7.5 containing 20% methanol; the other conditions are same as in Figure 2.

The concentration of chiral selectors is a parameter most optimized in enantioseparations. The influence of SBE_7_-β-CD concentration was investigated in the range of 5–50 mmol/L. Chiral resolution was achieved for all the concentrations within the range. With the increase of SBE_7_-β-CD concentration, a progressive separation of the enantiomers was observed (summarized in Figure 4). This might result from the following two aspects: (1) higher SBE_7_-β-CD concentration would reduce the apparent mobility, which would result in longer migration time and in turn longer interaction time of the analyte with the selector; (2) higher SBE_7_-β-CD concentration is beneficial to the complexation of the cyclodextrin with the analyte. However, as the SBE_7_-β-CD concentration increased from 20 mmol/L to 50 mmol/L, the resolution tendency becomes flat due to enhanced peak broadening, while that of migration time increase significantly (from 12.8 min to 29.4 min). A SBE_7_-β-CD concentration of 20 mmol/L was finally adopted as a compromise between resolution and run time.

#### 2.1.4. Effects of Temperature

**Figure 5:**
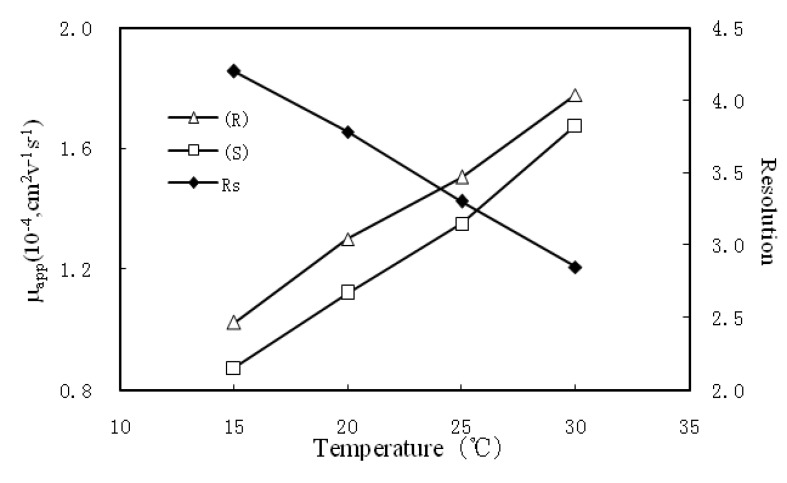
The effect of temperature on resolution. Separation condition: 20 mmol/L SBE_7_-β-CD in 20 mmol/L phosphate buffer (pH 7.5) containing 20% methanol; the other conditions are same as in Figure 2.

The influence of temperature was also considered in the optimization process of the CE separations. BGE viscosity and complex stability will vary with the change of temperature and thereby affect the run time and resolution. This effect was investigated at four temperatures (15, 20, 25 and 30 °C), and the result is illustrated in Figure 5. The enantioresolution decreased as the temperature increased with a shorter migration time. Considering both run time and resolution, the temperature of 25 °C is suitable for modafinil enantiomeric separation. Thus, the optimal separation method was 20 mmol/L SBE_7_-β-CD in 20 mmol/L phosphate buffer (pH 7.5) containing 20% methanol, with temperature set at 25 °C.

### 2.2. Method Validation

The optimal enantioseparation condition was subsequently used to quantitative determination of the enantiomeric impurity for armodafinil. The method validation was carried out as follows.

#### 2.2.1. Selectivity

**Figure 6:**
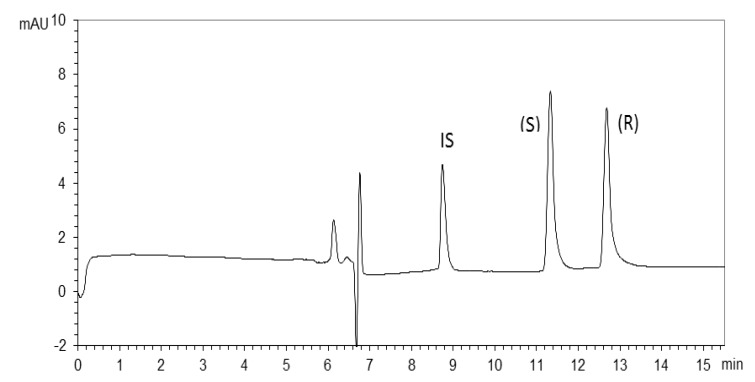
Electrophoretic separation of racemic modafinil at a concentration of 1.0 mg/mL. Separation condition: Benzamide (50 μg/mL) used as internal standard; 20 mmol/L SBE_7_-β-CD in 20 mmol/L phosphate buffer (pH7.5) containing 20% methanol; temperature, 25 °C; applied voltage, 20 kV; detection wavelength, 225 nm; injection, 50 mbar for 5 s; capillary, 50 cm × 50 μm i.d. with effective length of 41.5 cm.

Selectivity of the method was demonstrated by running standard solutions of armodafinil, (*S*)-modafinil and the racemates. By comparing the electropherograms of armodafinil and (*S*)-modafinil it is clear that (*S*)-modafinil migrates faster to the detector than armodafinil. To improve the repeatability of the proposed method, a solution of benzamide was used as an internal standard (IS). The electropherogram displayed in Figure 6 was obtained by running a mix solution of racemic modafinil (100 μg/mL) and IS benzamide (50 μg/mL) and it shows that a satisfactory resolution of armodafinil and its (*S*)-enantiomer was achieved.

#### 2.2.2. Repeatability

The repeatability of migration times and peak areas was investigated by six runs in succession of racemic modafinil at the concentration of 50 µg/mL. The RSD of migration times were 1.77% and 1.50% for (*S*)-modafinil and armodafinil, respectively. The RSD of peak area ratios of (*S*)-modafinil to IS and that of armodafinil to IS were 2.12% and 2.45%, respectively.

#### 2.2.3. LOD and LOQ

Signal-to-noise ratio of 3 and 10 are generally considered as limit of detection (LOD) and limit of quantification (LOQ), respectively. The LOD and LOQ of (*S*)-modafinil obtained by the method were 1.25 μg/mL and 2.50 μg/mL.

#### 2.2.4. Linearity

The linearity of (*S*)-modafinil was studied by preparing and assaying a series calibration standards at six different concentrations within the range from 5.0 μg/mL to 50 μg/mL. A good linear relationship was observed between the peak area ratios of (*S*)-modafinil to IS and the concentrations in the investigated concentration range. The linear equation is given out in Table 1.

**Table 1:** The linearity result of (*S*)-modafinil by the established method.

Conc.(μg/mL)	5	10	20	30	40	50
Peak area ratio	0.20	0.49	0.88	1.37	1.81	2.33
Calibration curve	A = 0.0463C − 0.0144					
	r = 0.9992					

#### 2.2.5. Accuracy and Precision

The investigation of accuracy was carried out with armodafinil samples (5 mg/mL) spiking (*S*)-modafinil at three concentration levels (5, 12.5, and 25 μg/mL). The test samples of each level were assayed in triplicate. The results (listed in Table 2) indicate that the accuracy of the proposed method for determination of the enantiomeric impurity determination of armodafinil was acceptable. 

**Table 2:** The accuracy result of armodafinil by the established method (n = 9).

Added conc.	Measured conc	Acurracy
μg/mL	(mean ± SD)(μg/mL)	(%)
5.0	4.85 ± 0.20	97.0
12.5	12.26 ± 0.43	98.1
25.0	24.38 ± 0.75	97.5

**Figure 7:**
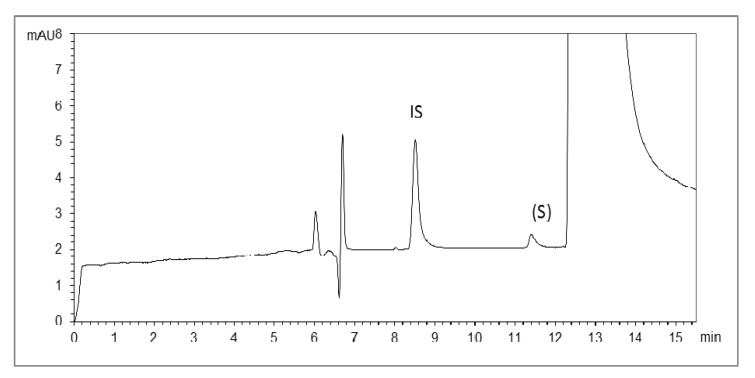
Armodafinil standard (5 mg/mL) containing 0.1% (*S*)-modafinil. Other conditions are as in Figure 6.

The test solution containing 0.1% enantiomeric impurity was used to evaluate precision by replicate testing for six times. The RSD values of peak area ratio of (*S*)-modafinil to IS and migration time of (*S*)-modafinil were 2.09% and 4.05%, Figure 7 respectively.

### 2.3. Application in Bulk Samples

The method was applied to determine the enantiomeric impurity of armodafinil in bulk samples. (*S*)-modafinil was not detected in a bulk sample of armodafinil. Thus, a sensitive and effective method of determining enantiomeric impurity at a concentration of 0.1% can be estimated so that the impurity control of armodafinil can be regarded as reliable.

## 3. Experimental

### 3.1. Reagents and Chemicals

Racemic modafinil, armodafinil standard, and armodafinil bulk sample with batch number of 20060406 were obtained from the Medicinal and Chemical Institute, China Pharmaceutical University (Nanjing, China). (*S*)-modafinil was purchased from Chemsky International Co., Ltd (Shanghai, China). SBE_7_-β-CD was kindly provided by Jiangsu Juhuan Pharmaceutical Co., Ltd. (Nanjing, China). β-CD, γ-CD, HP-β-CD, M-β-CD and HP-γ-CD were purchased from Cyclochem Chemicals Co., Ltd. (Kunshan, China). Methanol, acetonitrile and isopropanol (all of HPLC-grade) were purchased from Jiangsu Hanbon Sci. & Tech. Co., Ltd. (Nanjing, China). Benzamide, disodium hydrogen phosphate and phosphoric acid were analytical grade and obtained from Nanjing Chemical Reagent Co. (Nanjing, China). Double distilled water was used throughout the study. The stock solutions of racemic modafinil, (*S*)-modadinil and armodafinil were prepared in methanol at a concentration of 1 mg/mL and working solutions were prepared by diluting the stock solutions with water. The stock solution of benzamide (1 mg/mL) was prepared in methanol used as the internal standard of the assay.

### 3.2. Instrumentation

All capillary electrophoresis experiments were carried on an Agilent 3D capillary electrophoresis system from Agilent Technologies (Waldbronn, Germany), equipped with a sampling device, a power supply, a temperature control and diode array detection (wavelength range from 190 to 600 nm). The system was controlled by the HP^3D^ CE ChemStation Software. Separation was performed in an untreated 50 μm I.D. fused-silica capillary with a total length of 50 cm and an effective length of 41.5 cm (Yongnian Optical Fiber Factory, Yongnian, Hebei, China).

### 3.3. Analysis

A new capillary was conditioned by flushing with 1 mol/L sodium hydroxide for 30 min, followed by water and 0.1 mol/L sodium hydroxide each for 30 min, and then rinsed with BGE for 10 min before it was used for CE analysis for the first time. At the end of each day, the capillary was flushed successively with 0.1 mol/L sodium hydroxide and water for 5 min, respectively. In order to obtain good peak shapes and repeatable migration data, the capillary was rinsed with 0.1 mol/L sodium hydroxide, water and BGE for 3 min between each runs.

Sample introduction was performed by pressure injection at 50 mbar for 5 s. The detection wavelength was set at 225 nm. The capillary temperature was optimized in the range of 15~30 °C. A constant voltage of 20 kV was applied during the separation. 

BGEs containing disodium phosphate, organic modifier and SBE_7_-β-CD or other CDs were used in this study. The phosphate solution was prepared by dissolving the appropriate amount of disodium hydrogen phosphate in water to a concentration of 20 mmol/L and adjusting the pH to a desired value with 20% (w/v) phosphoric acid solution or 1 mol/L sodium hydroxide. BGEs were then got by dissolving a corresponding amount of SBE_7_-β-CD and adding that of organic modifier into the above disodium phosphate solution. BGEs were investigated by varying the pH value in the range of 6.0~8.0, and varying the chiral selector in the range of 5~50 mmol/L and the organic modifier in the range of 10–40% (v/v).

### 3.4. Electrophoretic Parameters

The resolution factor (Rs) was calculated by the formula R_S_ = 2 (*t*_2_ - *t*_1_)/(w_1_ + w_2_), Where *t*_1_ and *t*_2_ are the migration times of the two enantiomers, and w_1_ and w_2_ are the peak widths. The apparent mobility of the enantiomers was calculated as *µ_app_* = *LI*/*Vt*, where *L* is the total length of the capillary (cm), *I* is the effective length of the capillary (cm), *V*is the applied voltage (V) and *t* is the migration time (s).

## 4. Conclusions

An enantioselective capillary electrophoresis method was established for the determination of the enantiomeric impurity of armodafinil. The resolution of the two enantiomers is satisfactory, and the enantiomeric impurity (*S*)-modafinil migrates before the main enantiomer armodafinil, which makes the method useful for the enantiomeric purity control of armodafinil. Furthermore, the proposed method was proven by method validation to be characterized with good repeatability, linearity, accuracy and low limit of detection. Finally, the developed method was successfully applied to the enantiomeric impurity determination of (*S*)-modafinil in an armodafinil bulk sample.
